# Sensitivity of cervical cytology in endometrial cancer detection in a tertiary hospital in Spain

**DOI:** 10.1002/cam4.4217

**Published:** 2021-09-04

**Authors:** Jon Frias‐Gomez, Eva Tovar, August Vidal, Lluis Murgui, Raquel Ibáñez, Paula Peremiquel‐Trillas, Sonia Paytubi, Nuria Baixeras, Alba Zanca, Jordi Ponce, Marta Pineda, Joan Brunet, Silvia de Sanjosé, Francesc Xavier Bosch, Xavier Matias‐Guiu, Laia Alemany, Laura Costas, Screenwide Team

**Affiliations:** ^1^ Cancer Epidemiology Research Programme IDIBELL Catalan Institute of Oncology Barcelona Spain; ^2^ University of Barcelona (UB) Barcelona Spain; ^3^ Information Technologies Department Hospital Universitari de Bellvitge IDIBELL Barcelona Spain; ^4^ Consortium for Biomedical Research in Oncology (CIBERONC) Madrid Spain; ^5^ Consortium for Biomedical Research in Epidemiology and Public Health (CIBERESP) Madrid Spain; ^6^ Department of Pathology Hospital Universitari de Bellvitge IDIBELL Barcelona Spain; ^7^ Department of Gynecology Hospital Universitari de Bellvitge IDIBELL Barcelona Spain; ^8^ Hereditary Cancer Program IDIBELL Catalan Institute of Oncology Barcelona Spain; ^9^ Medical Sciences Department School of Medicine University of Girona Girona Spain; ^10^ National Cancer Institute Rockville Washington USA; ^11^ Universitat Oberta de Catalunya (UOC) Barcelona Spain

**Keywords:** cervical cytology, cervico‐vaginal cytology, endometrial cancer, sensitivity

## Abstract

**Introduction:**

Cervical cytology is a well‐stablished cervical cancer screening method. However, due to the anatomical continuity of the genital tract, it can also detect signs of endometrial disease. Our aim was to estimate the sensitivity of cervical cytology in endometrial cancer detection and prognosis in a large population over a 30‐year period in a large academic tertiary hospital in Spain.

**Methodology:**

We performed a search for women diagnosed with endometrial cancer from 1990 to 2020, who were surgically treated and had a previous cervical cytology result. Information Technologies Department databases from Bellvitge University Hospital and the Screenwide case–control study's database were used. Cervical cytology results were classified as abnormal when squamous lesions, glandular atypia or malignant cells were identified.

**Results:**

Overall, we evaluated 371 women with endometrial cancer and a documented cervical cytology performed within 3 years previous to surgical treatment. Overall, the sensitivity of cervical cytology for endometrial cancer detection was 25.6%. Several clinico‐pathological characteristics, such as non‐endometrioid histology and a higher stage, were correlated with higher sensitivity.

**Discussion:**

We observed a low sensitivity of cervical cytology to effectively diagnose endometrial cancer. However, recent technological advances using genomics and epigenomics may offer a promising perspective to detect endometrial cancer with high sensitivity in these cervical specimens.

## Graphical abstract

Cervical cytology sensitivity for endometrial cancer was 25.6%. Several clinico‐pathological factors, such as non‐endometrioid histology and a higher stage, were correlated with higher sensitivity.

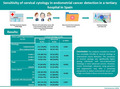

## 1 INTRODUCTION

In 2018, endometrial cancer (EC) was one of the most common gynaecological cancers which accounted for 8.4 cases per 100,000 women worldwide.^1^ EC has been classically classified into two groups: type I (endometrioid), the most common one, which usually shows a good prognosis and type II (non‐endometrioid), a less common heterogeneous subtype (10%–15%), which shows a poor prognosis.^2^ Although the dualistic classification system is widely used, a new classification system proposed by The Cancer Genome Atlas (TCGA) Consortium based on molecular features is starting to be integrated into clinical practise.^3^, ^4^ Due to the expected rise in obesity rates and ageing of the population, the EC burden is likely to increase over the next years.^2^, ^5^

Cervical cytology is a well‐stablished test in cervical cancer screening programmes, which have helped to reach a large reduction in cervical cancer incidence and mortality.^6^, ^7^ The anatomical continuity of the uterine cavity with the cervix makes the cervical cytology also accessible to evaluate signs of disease shed from the endometrium. Therefore, collecting cervical cells may also allow sampling of abnormal endometrial cells, if any. Likewise, several retrospective studies have shown abnormal cells of suspected endometrial origin at cervical cytological findings in 45% of cases with EC on average.^8^ Nevertheless, recent molecular advances in the genomic, epigenomic and proteomic exploitation of these samples could offer a benefit in the early detection of EC, with sensitivities up to ≈80%.^9^ We performed a retrospective evaluation to examine the sensitivity of routine cervical cytology among women with EC in a large academic tertiary hospital in Spain between 1990 and 2020.

## 2 MATERIALS AND METHODS

The study was based on women with EC diagnosed in different time periods in the Bellvitge University Hospital identified using three databases: two retrospective databases from the Information and Technologies Department (1990–2018) and a data set from the Screenwide case–control study (2017–2020). Databases from the Information and Technologies Department include automatically extracted data collected in the electronic medical pathological files of patients. Data from the case–control study are extracted individually from the participants' medical files by an experienced investigator. All data are linked using the regional medical identification number. The Bellvitge University Hospital is a reference tertiary hospital for more than 1,800,000 people and more than 2,500,000 people for high‐grade EC referrals.

Women surgically treated for EC and who had previous cervical cytology performed within 3 years from the hysterectomy date were identified in the two retrospective databases: one contained data from 1990 to 2014 and another one with data from 2014 to 2018. Data were also extracted from the Screenwide case–control study in which consecutive women with a confirmed diagnosis of incident EC were prospectively enrolled from 2017 to 2020 and pre‐operative cervical cytology was collected. Extracted data from the electronic medical records included histology, grade, stage and presence of symptoms (only for prospective cases), date of hysterectomy, tumour extent and lymphatic affection when available. We also extracted cytology results and the dates on which cervical cytology were performed. If more than one cervical cytology was performed within the selected period, the most recent cytology before the hysterectomy was selected. We excluded cases with cervical cytology performed uniquely before 3 years from the hysterectomy date and those with unknown dates of hysterectomy (Figure S1).

Conventional Pap tests were used up to 2010 and liquid‐based (ThinPrep^®^) afterwards. Cervical cytology diagnoses were recoded as normal versus abnormal. Abnormal diagnoses included: (1) squamous lesions (atypical squamous cells of undetermined significance [ASCUS], low‐grade squamous intra‐epithelial lesions [LSIL], high‐grade squamous intra‐epithelial lesions [HSIL] and atypical squamous cells cannot exclude HSIL [ASC‐H]); (2) glandular atypia, including atypical glandular cells of undetermined significance (AGUS) or (3) malignant cells. Bethesda system to classify cytology results was used in our centre from 2000 onwards. We categorised as pre‐operative those cervical cytology performed within 2 months from the intervention date. We estimated the overall sensitivity as the number of women with true‐positive results (abnormal cervical cytology results) divided by the number of women with endometrial cancer (true positives + false negatives). We analysed results by several factors including histology and tumour extent. We applied Fisher's exact tests for proportions to compare several clinico‐pathological characteristics. Confidence intervals (CIs) were calculated using a binomial exact test (‐cii‐ command in Stata). All statistical analyses were performed using Stata v.16.^10^

## 3 RESULTS

Between 1990 and 2020, we identified 1521 women diagnosed with EC at the Bellvitge University Hospital. Three hundred and seventy‐one of them (24.4%) had a documented cervical cytology performed within 3 years from the hysterectomy date. Included women had similar histology than women without a documented Pap within 3 years from a hysterectomy (*p* value >.05), although they were younger at diagnosis (*p* value <.01). The age of patients ranged between 28 and 91 years old, and 96.8% were older than 45 years. An abnormal cervical cytology result was observed in 25.6% (95% CI: 21.2%–30.4%) (*N* = 95) of included women. Of them, 48 had malignant cells, 41 displayed atypical glandular cells including AGUS and 6 had squamous lesions (Table 1). No statistically significant differences were observed between results from retrospective and prospective data sets (24.6% vs. 26.9%, respectively; *p* value = .633) (Table S1).

**TABLE 1:** Findings in patients with endometrial cancer and cervical cytology performed within 3 years previous to surgical treatment

Cervical cytology results	*N* (%)	[95% CI]
Normal	276 (74.4%)	[69.6%−78.8%]
Abnormal	95 (25.6%)	[21.2%−30.4%]
Squamous lesions^a^	6 (1.6%)	[1.0%−3.5%]
Glandular atypia (including AGUS)	41 (11.1%)	[8.0%−14.7%]
Malignant	48 (12.9%)	[9.7%−16.8%]

Abbreviations: AGUS, atypical glandular cells of undetermined significance; CI, confidence interval.

^a^ Squamous lesions include: atypical squamous cells of undetermined significance (ASCUS), low‐grade squamous intraepithelial lesions (LSIL), high‐grade squamous intraepithelial lesions (HSIL) and atypical squamous cells, cannot exclude HSIL (ASC‐H).

Sensitivity was higher for non‐endometrioid histology compared with endometrioid cancers (37.3% vs. 21.2%, *p* value = .002, Table 2). Statistically significant differences were observed according to grades (11.8%, 32.7% and 34.8% for Grades 1, 2 and 3, respectively, *p* value <.001). Likewise, sensitivity was higher for those with advanced stages (II/III/IV) in comparison with stage I (55.0% vs. 16.2%, *p* value <.001). Statistically significant differences were also observed for tumour extent (16.2%, 68.0% and 38.3% for tumour extent T1, T2 and T3, respectively, *p* value <.001) and lymphatic affection (20.8% and 43.8% for N0 and N1–2, respectively, *p* value = .007). No statistically significant differences were observed according to the time period of diagnosis (*p* value = .895). Most of the cytology were performed pre‐operatively: 58.4% of the cytology were performed within ≤2 months from hysterectomy. We found statistically significant differences by time from hysterectomy (31.6% vs. 17.6%, for cytology performed within ≤2 months and >2 months from hysterectomy, respectively, *p* value = .003, Table 2). No statistically significant differences were observed according to the presence of symptoms (*p* value = .798, Table S2).

**TABLE 2:** Sensitivity of cervical cytology for endometrial cancer detection among 371 women, stratified by clinico‐pathological characteristics

Clinico‐pathological variables *N* = 371	Cervical cytology results		*p* value^b^
	Normal *N* (%)	Abnormal^a^ *N* (%)	
Histology			
Endometrioid	212 (78.8%)	57 (21.2%)	.002
Non‐endometrioid	64 (62.8%)	38 (37.3%)	
Grade			
Grade 1	134 (88.1%)	18 (11.8%)	<.001
Grade 2	33 (67.3%)	16 (32.7%)	
Grade 3	76 (65.5%)	40 (34.8%)	
Stage^c^			
I	93 (83.8%)	18 (16.2%)	<.001^b^
II/III/IV	18 (45.0%)	22 (55.0%)	
Tumour extent			
T1	218 (83.9%)	42 (16.2%)	<.001^b^
T2	8 (32.0%)	17 (68.0%)	
T3	29 (61.7%)	18 (38.3%)	
Lymphatic affection (node)			
N0	183 (79.2%)	48 (20.8%)	.007
N1 and N2	18 (56.3%)	14 (43.8%)	
Time period of diagnosis			
1990–2009	95 (75.4%)	31 (24.6%)	.895
2010–2017	66 (75.0%)	22 (25.0%)	
2018–2020	105 (72.9%)	39 (27.1%)	
Time lag between cervical cytology and hysterectomy			
≤2 months	145 (68.4%)	67 (31.6%)	.003
>2 months	131 (82.4%)	28 (17.6%)	

Note

Numbers do not always add up due to missing data.

Abbreviation: IQR, inter‐quartile range.

^a^ Abnormal Pap results include squamous lesions, atypical glandular cells of undetermined significance (AGUS), atypia and malignant lesions.

^b^ Fisher's exact test.

^c^ Data available only for the prospective study.

## 4 DISCUSSION

Cervical cytology is a well‐stablished tool that has allowed the implementation of effective cervical screening programmes and has contributed to achieve a great reduction of incidence and mortality.^6^, ^7^ Consequently, George Papanicolaou, the inventor of the Pap test, became interested at the end of his career in the diagnostic value of cervico‐vaginal smears in the detection of EC. Unfortunately, he acknowledged that the cytology was not equally satisfactory for EC detection compared with cervical malignancies, and therefore, it has never been proposed as a screening approach neither a down‐staging approach until early tumour molecular markers are being identified in cervical scrapes.^11^, ^12^

We observed that only 25.6% of women with EC had abnormal cervical cytology before hysterectomy. Around half of these lesions already suggested a malignancy. The sensitivity of cervical cytology was higher for non‐endometrioid histology and more advanced cancers but still with low performance. Sensitivity to detect EC observed in this study was low, although results may be relevant considering the transition in several regions from cytology‐based to primary HPV testing in cervical cancer screening programmes. A recent systematic review of the literature showed that 45% of women with EC had an abnormal Pap before surgery or diagnosis.^8^ However, Castle et al., with a large sample size (*n* = 3414 EC cases), observed recently that only 12.65% of EC were preceded by abnormal cytology.^13^ While some of the studies included in the meta‐analyses referred to pre‐operative cytology, Castle et al. included routine cytology for cervical cancer screening, which could contribute to the observed low sensitivity in this study. We searched for all cytology in the Bellvitge University Hospital and observed that 17.6% of cytology performed >2 months from hysterectomy were abnormal, which is in line with Castle et al., findings. Other factors could have also contributed to the observed low sensitivity in our study. We did not observe normal endometrial cells among post‐menopausal women in our centre. Analyses restricted to atypical and malignant cells in this systematic review revealed a sensitivity of 44%,^8^ which is still higher to that observed in our study. A long debate exists on the report of benign‐appearing endometrial cells, and 2014 Bethesda system^14^ recommends reporting normal endometrial cells among women 45 years or older, although this cut‐off age is still under debate.^15^ On the other hand, the pathologist knowledge on the EC status may also have influenced results, but unfortunately, we cannot evaluate this information.

To our knowledge, this is the study evaluating the longest period of time (1990–2020), and one of the largest analyses on the topic. Altogether, this allows a reasonable statistical power to evaluate the sensitivity of cervical cytology in EC detection. The centre covers a large population of the region, although it may treat more advanced cancers in comparison to other hospitals, which could result in an over‐estimation of the sensitivity of cervical cytology for EC. However, we observed a relatively low sensitivity of cervical cytology in EC. This may be in part because women who did not undergo surgery were not included. These women may have more advanced cancers and therefore may be more likely to have abnormal cytology results. Part of the data was retrospective, and the databases were not designed for this study purpose. Retrospective data, in particular oldest data, may have misclassification issues due to the lack of reports from some pathologists or certain variables. For instance, retrospective databases did not contain information on tumour spread, and therefore, analysis on stage was restricted to prospective data. Prospective data may be more reliable, although we did not observe statistically significant differences in sensitivity between retrospective and prospective data sets.

In conclusion, our analyses revealed an overall low sensitivity of cervical cytology in EC detection. The sensitivity of cervical cytology was significantly higher among non‐endometrioid histology compared with endometrioid histology. Similarly, other clinico‐pathological characteristics related to a high stage of the tumour were also associated with significantly higher sensitivity. Contrary, sensitivity was lower when cervical cytology were performed >2 months from hysterectomy, rather than pre‐operatively. Based on our estimates, the current sensitivity of the cervical cytology is too low to effectively diagnose EC, although recent technological advances using genomics and epigenomics may offer a promising perspective to detect EC with a higher sensitivity in these cervical specimens.

## CONFLICT OF INTEREST

Authors do not have conflicts of interest to declare.

## ETHICAL APPROVAL

PR281/16 CEIC HUB 14/3/2019.

## INFORMED CONSENT

For the retrospective databases, patients' informed consent is an exception as they involve large data sets, are retrospective and observational, characteristics that make consent impractical to collect. For the prospective case–control study database, all eligible subjects signed an informed consent form after receiving information from the study and before any intervention. In both scenarios, security measures are taken accordingly in order to protect patient confidentiality.

## Supporting information

Supplementary Material

## Data Availability

Data available on request due to privacy/ethical restrictions.
