# Supplementary material for: Sensitivity of cervical cytology in endometrial cancer detection in a tertiary hospital in Spain
Source: Cancer Med. 2021 Sep 4;10(19):6762–6. doi: 10.1002/cam4.4217 (PMC8495290; doi:10.1002/cam4.4217)
Supplement: Supplementary file 1 — Supplementary Material [file CAM4-10-6762-s001.docx]

**SUPPLEMENTARY MATERIAL**

**Sensitivity of cervical cytology in endometrial cancer detection in a tertiary hospital in Spain**

**Supplemental Table 1.** Findings in patients with endometrial cancer and a cervical cytology performed within 3 years previous to surgical treatment according to data collection (retrospective vs. prospective)

**Supplemental Table 2.** Sensitivity of cervical cytology for endometrial cancer detection among 160 women, according to the presence of symptoms in the prospective study

**Supplemental Figure 1.** Flow chart

**Supplemental Table 1.** Findings in patients with endometrial cancer and a cervical cytology performed within 3 years previous to surgical treatment according to data collection (retrospective vs. prospective)

|  | **Retrospective**  **(1990-2018) N=211** | | | | **Prospective**  **(2017-2020) N=160** | |
| --- | --- | --- | --- | --- | --- | --- |
| **Cervical cytology result** | **N (%)** | | **[95% CI]** | | **N (%)** | **[95% CI]** |
| **Normal**  **Abnormal**  **Squamous lesions^a^**  **Glandular atypia (including AGUS)**  **Malignant** | | 159 (75.4%)  52 (24.6%)  4 (1.9%)  13 (6.2%)  35 (16.6%) | | [69.0%-81.0%]  [19.0%-31.0%]  [1.0%-4.8%]  [3.3%-10.3%]  [11.8%-22.3%] | 117 (73.1%)  43 (26.9%)  2 (1.3%)  28 (17.5%)  13 (8.1%) | [65.6%-79.8%]  [20.2%-34.4%]  [0.2%-4.4%]  [12.0%-24.3%]  [4.4%-13.5%] |
| CI: Confidence interval; AGUS: atypical glandular cells of undetermined significance.  ^a^ Squamous lesions includes: Atypical Squamous cells of undetermined significance (ASCUS), low-grade squamous intraepithelial lesions (LSIL), high-grade squamous intraepithelial lesions (HSIL) and atypical squamous cells, cannot exclude HSIL (ASC-H). | | | | | | |

**Supplemental Table 2.** Sensitivity of cervical cytology for endometrial cancer detection among 160 women, according to the presence of symptoms in the prospective study

| **Abnormal bleeding** | **Cervical cytology results** | |  |
| --- | --- | --- | --- |
|  | **Normal** | **Abnormal^a^** | **p-value^b^** |
|  | **N (%)** | **N (%)** |  |
|  |  |  |  |
| **No** | 15 (71.4%) | 6 (28.6%) | 0.798 |
| **Yes** | 102 (73.4%) | 37 (26.6%) |  |
| ^a^ Abnormal Pap results include squamous lesions, atypical glandular cells of undetermined significance (AGUS), atypia and malignant lesions. | | | |
| ^b^ Fisher’s exact test. | | | |

**Supplemental Figure 1.** Flow chart

| **A) Retrospective database 1990-2014:**  1031 endometrial cancer cases  424 without cervical cytology  608 endometrial cancer cases with cervical cytology  41 date of hysterectomy missing  567 endometrial cancer cases with cervical cytology and histological confirmation  323 cervical cytologies performed after to after hysterectomy  6 cervical cytologies performed > 3 years from hysterectomy  8 without cervical cytology results  176 cases | **B) Retrospective database 2014-2018:**  328 endometrial cancer cases  82 date of hysterectomy missing  246 endometrial cancer cases with hysterectomy  43 without cervical cytology  131 cervical cytologies performed after to after histological confirmation  1 cervical cytology > 3 years from histological confirmation  36 cases identified in the prospective study  35 cases | **C) Prospective study 2017-2020:**  187 endometrial cancer cases were eligible for the study  15 declined to participate in the study  172 endometrial cancer cases enrolled in the study  12 without cervical cytology  160 cases |
| --- | --- | --- |
